# Alteration of hepatocellular antioxidant gene expression pattern and biomarkers of oxidative damage in diazinon-induced acute toxicity in Wistar rat: A time-course mechanistic study

**DOI:** 10.17179/excli2017-760

**Published:** 2018-01-08

**Authors:** Shokoufeh Hassani, Faheem Maqbool, Armin Salek-Maghsoudi, Soheila Rahmani, Amir Shadboorestan, Amir Nili-Ahmadabadi, Mohsen Amini, Parviz Norouzi, Mohammad Abdollahi

**Affiliations:** 1 Toxicology and Diseases Group, Pharmaceutical Sciences Research Center, Tehran University of Medical Sciences, Tehran, Iran; 2 Department of Toxicology and Pharmacology, Faculty of Pharmacy, Tehran University of Medical Sciences, Tehran, Iran; 3 Department of Pharmacology and Toxicology, School of Pharmacy, Hamadan University of Medical Sciences, Hamadan, Iran; 4 Cancer Therapy Group, Pharmaceutical Sciences Research Center, and Department of Medicinal Chemistry, Faculty of Pharmacy, Tehran University of Medical Sciences, Tehran, Iran; 5 Center of Excellence in Electrochemistry, Faculty of Chemistry, University of Tehran, Tehran, Iran; 6 Endocrinology and Metabolism Research Institute, Tehran University of Medical Sciences, Tehran, Iran

**Keywords:** biomarker, diazinon, gene expression, molecular toxicity, HPLC, oxidative stress, pesticide

## Abstract

In the present survey, the plasma level of diazinon after acute exposure was measured by HPLC method at a time-course manner. In addition, the impact of diazinon on the expression of the key genes responsible for hepatocellular antioxidative defense, including PON1, GPx and CAT were investigated. The increase in oxidative damages in treated rats was determined by measuring LPO, protein carbonyl content and total antioxidant power in plasma. After administration of 85 mg/kg diazinon in ten groups of male *Wistar* rats at different time points between 0-24 hours, the activity of AChE enzyme was inhibited to about 77.94 %. Significant increases in carbonyl groups and LPO after 0.75 and 1 hours were also observed while the plasma antioxidant power was significantly decreased. Despite the dramatic reduction of GP_X_ and PON1 gene expression, CAT gene was significantly upregulated in mRNA level by 1.1 fold after 4 hours and 1.5-fold after 24 hours due to diazinon exposure, compared to control group. Furthermore, no significant changes in diazinon plasma levels were found after 4 hours in the treated rats. The limits of detection and quantification were 137.42 and 416.52 ng/mL, respectively. The average percentage recoveries from plasma were between 90.62 % and 95.72 %. In conclusion, acute exposure to diazinon increased oxidative stress markers in a time-dependent manner and the changes were consistent with effects on hepatic antioxidant gene expression pattern. The effect of diazinon even as a non-lethal dose was induced on the gene expression of antioxidant enzymes. The change in antioxidant defense system occurs prior to diazinon plasma peak time. These results provide biochemical and molecular evidence supporting potential acute toxicity of diazinon and is beneficial in the evaluation of acute toxicity of other organophosphorus pesticides as well.

## Introduction

Organophosphorus (OP) pesticides are used in large amounts and considered as one of the most serious environmental contaminants of today's world (Mostafalou and Abdollahi, 2013[36]). These chemicals are used to control purposeless growth of pests involved in spreading various diseases as the vector. Factors which are involved in the intensity of its toxic effects include dose, route of exposure, percent of absorption, and physicochemical properties of different types of OPs. The rate of detoxification as well as duration of toxicity play important roles (Abdollahi et al., 2004[2]; Mostafalou et al., 2013[39]). There is clear evidence in the literature about the link between different pesticides exposure and occurrence of various human chronic diseases, such as cancer, Parkinson, Alzheimer, multiple sclerosis, diabetes, cardiovascular and chronic kidney diseases (Mostafalou and Abdollahi, 2017[37]; Souza et al., 2011[56]; Mostafalou et al., 2012[38]; Maqbool et al., 2016[30], 2017[31]; Pakravan et al., 2016[44]). Diazinon (O,O-diethyl O-[4-methyl-6-(propan-2-yl) pyrimidin-2-yl] phosphorothioate) (DZN) is an OP insecticide which is extensively used in agriculture and households (Pakzad et al., 2013[45]). Although persistence of DZN in the environment is very low, the residual amounts are highly toxic for humans and animals (Khoshbavar-Rostami et al., 2006[24]; Shah and Iqbal, 2010[52]; Zhou et al., 2011[61]). The detection of pesticides is extremely essential in order to prevent their cellular damage. Currently various analytical techniques utilized to detect the pesticides include high-performance liquid chromatography (HPLC) (García-Valcárcel and Tadeo, 2009[17]), gas chromatography-mass spectrometry (GC–MS), capillary electrophoresis (Regueiro et al., 2015[49]), fluorometry (Pacioni and Veglia, 2003[43]), and biosensors (Hassani et al., 2017[20]). It is interesting to note that, like other OPs, the most common and familiar mechanism of toxicity of DZN is the inhibition of acetyl cholinesterase (AChE) enzyme by phosphorylation of residues such as serine in the active site of the enzyme in the target tissues (Nili-Ahmadabadi et al., 2013[40]). DZN is metabolized in the body through oxidative phosphorylation being still potent AChE inhibitor along with neurotoxic effects (Colovic et al., 2010[12]; Shah & Iqbal, 2010[52]). Besides inhibition of AChE, production of free radicals, induction of oxidative stress is an important mechanism that should not be forgotten (Soltaninejad and Abdollahi, 2009[55]; Bhatti et al., 2010[9]; Lukaszewicz-Hussain, 2010[28]). The antioxidant defense system is comprised of different enzymes like superoxide dismutase (SOD), catalase (CAT) and glutathione S-transferase (GST), whereas non-enzymatic antioxidants include vitamins (vitamins C and E), ß-carotene, and reduced glutathione (GSH). Both of these series protect the cells from oxidative damage (Kose et al., 2010[25]; Shokrzadeh et al., 2013[54]). Paraoxonases (PON’s) are a group of enzymes with different subtypes involved in antioxidant defense mechanisms in different tissues/organs of the body. PON1 gene expression takes place in the liver, where is involved in hydrolyzing of toxic metabolites and functioning against lipid oxidation toward the defense system, especially in exposure to different OPs (Bhatti et al., 2010[9]). Simply, it can be stated that oxidative stress is an imbalance between the rate of reactive oxygen species (ROS) production and the protective capacity of antioxidants (Monteiro et al., 2009[35]; Ojha et al., 2011[42]).

Previous reports have explained molecular and biochemical pathways involved in chronic toxicity of DZN in different organs including liver, kidney and pancreas (Shadboorestan et al., 2016[51]; Shiri et al., 2016[53]; Khaksar et al., 2017[23]), however, there are some inconsistencies in the literature on the toxicity of DZN in acute exposure and thus its exact mechanisms are not fully elucidated.

The main aim of the present study was to evaluate the time-dependent acute changes in DZN plasma level along with expression of the key genes responsible for hepatocellular anti-oxidative defense. 

## Materials and Methods

### Chemicals

Diazinon standard (> 98 %) from Supelco Company (USA), di-nitrophenyl-hydrazene (DNPH), acetonitrile (ACN, HPLC grade) from Caledon (Canada), solid phase extraction (SPE) cartridge octadecyl (C18) from Teknokroma (Spain), trichloro acetic acid (TCA) and TRIzol reagent from Roche (Swiss), tripure isolation reagent and expand reverse transcriptase from Roche Applied Sciences (Germany), tris-HCl buffer, FeCl_3_, KCL, MgCl_2_, NaH_2_PO_4_, EDTA, glucose, sucrose from Merck (Germany), technical DZN from Shimi-Keshavarz Pesticide Production Co. (Iran), primers from Gen Fanavaran Co. (Iran), and SYBR^®^ Premix Ex Taq from Takara Bio Inc. (South Korea) were used in this study.

### Animals

Male* Wistar* rats (200-250 g), ranging from age of 2-3 months from the animal house of the TUMS were acquired 7 days prior to use. Propylene cages were used to house at a temperature of 21-25 °C, light cycle (12 h dark/12 h light) and humidity of 50 %. The water and also food were provided ad libitum.

The protocol of the study was approved by the institute ethical committee under code number IR.TUMS.VCR.REC.1395.1129 and all ethical issues on the use of animals were followed. 

### Selection of optimum dose 

The optimum dose of DZN was determined as 85 mg/kg, which is the same as 1/5th of its LD_50_, (350 mg/kg of body weight for rat) from commercial grade on the basis of pilot studies. The optimum dose of DZN was designed according to ~70 % inhibition of AChE and an increase of lipid peroxidation (LPO) in plasma samples (WHO, 2010[60]). 

### Animal treatment

According to the pilot study, the optimum dose of DZN was determined 85 mg/kg. The rats were distributed into ten groups, containing six animals each. DZN was dissolved in the corn oil and all groups received 85 mg/kg single dose of DZN via gavage, while the control group was treated with equal amount of the vehicle (corn oil). The plasma samples were collected after 0.25, 0.5, 0.75, 1, 2, 4, 6, 8, 12 and 24 hrs post DZN administration.

### Sample preparation

At the end of treatment, animals were anesthetized, using ketamine: xylazine (80-100 mg/kg: 5-10 mg/kg IP). Blood was rapidly taken from the heart. The plasma samples were obtained by means of centrifugation at 4000 g for 10 min in an EDTA test tube and stored at -80 °C until biomarker analysis. Liver tissue was taken after 4 and 24 hrs. The tissues were weighed first and kept in 10 mL of formalin 10 %, as a fixator for further histopathological evaluations. The second set of liver tissues after 24 hrs was then washed, using cold and sterile phosphate buffer (pH 7.4) and frozen at -80 °C for further molecular analysis.

### Determination of acetyl cholinesterase (AChE) activity in plasma

The change in color gave an indication of formation of thiocholine. Changes in absorbance were detected at 412 nm and the enzyme activity was represented as μg/mL. The already set up method was followed (Fakhri-Bafghi et al*.*, 2016[16]).

### Determination of oxidative stressbiomarkers

#### Measurement of lipid peroxidation (LPO) 

The final product of lipid peroxidation is malondialdehyde (MDA) that upon reacting with thiobarbituric acid (TBA) procreates a new complex named as TBA reactive substances (TBARS). TBARS were measured by absorbance recorded at 532 nm spectrophotometer (Armstrong and Browne, 1994[5]). By application of a standard curve such as 1,1´,3,3´-tetraethoxypropane, the amount of MDA formed in each sample was reported as μM/mg protein as detailed previously (Astaneie et al., 2005[6]).

#### Determination of total thiol molecules (TTM) 

Thiols containing sulfhydryl group were determined by the method of Hu (1994[21]). The interaction of TTM with DTNB gives a highly colored anion that gives a maximum peak at 412 nm determined by spectrophotometer. The already set up method was followed (Mohammadi et al*.*, 2011[34]).

#### Determination of ferric reducing/antioxidant power (FRAP) 

The FRAP test indicates the power of plasma in deoxidizing the Fe^3^+‏ to Fe^2^+.‏ The complex between TPTZ‏ and Fe^2^+ gives a blue color that its absorbance at 593 nm is measured by a spectrophotometer (Benzie and Strain, 1996[8]). The results were calculated as millimole per liter as previously set up (Abdolghaffari et al., 2010[1]).

#### Determination of protein carbonyl groups 

The oxidative protein damage was spectrophotometrically measured at 370 nm by determination of carbonyl groups (Levine et al., 1990[26]). Detailed procedure and different steps of the method were followed as set up and published previously (Bahadar et al*.*, 2015[7]).

### Protein estimation

Bradford method was used for protein quantitation that is based on an absorbance shift of the dye Coomassie Brilliant Blue G-250. Under acidic conditions the red form of the dye is converted into its bluer form and thus binding to the protein is assayed (Bradford, 1976[10]). As set up for this work, different concentrations of BSA, were used to draw standard curve between 0.25 and 1 mg/mL. The absorbance of the sample was measured at 595 nm, using a spectrophotometer (Moeini-Nodeh et al., 2017[33]). 

### Histopathological studies

The animals were euthanized 4 and 24 hrs post-treatment and their liver was isolated and fixed in the 10 % neutral buffered formalin (NBF, pH 7.26) for 48 hrs, and then embedded in paraffin. The 5 µm thick sections were made ready for staining with hematoxylin and eosin (H&E). The histological slides were evaluated using light microscopy (Olympus BX51, Japan). Any changes, including acute and chronic inflammatory response, fatty change, coagulative necrosis, hemorrhage or hyperemia and etc. were assessed in different samples.

### Determination of diazinon in plasma by HPLC instrumentation

The HPLC system (Waters, USA) consisted of Waters 510 pump and Waters 486 UV detector was used. Chromatography was conducted on 4.6 × 150 mm, 5 μm ZORBAX C18 column (Agilent, Germany). The sample cleanup was carried out via SPE column, C18/17 %, 3 mL/200 mg. 

### Sample preparation

For sample preparation, 0.2 mL of plasma from the untreated rats was collected between 200 to 6400 ng/mL each containing DZN. All samples were mixed with 1 M acetic acid (pH 5.0) and prepared for further analysis as described before (Abu-Qare and Abou-Donia, 2001[3]).

### Conditions for chromatography

A prepared plasma residue of 20 µL was injected into the HPLC. The isocratic mobile phase consisted of acetonitrile-water (80:20, v/v). The flow rate of the mobile phase was 1.0 mL/min, which was freshly prepared before each run and was degassed for 10 minutes by ultrasonication. The eluents were examined by the UV detection of the wavelengths 254 nm for DZN and the chromatographic analysis was done at ambient conditions. 

### Calibration procedures

Six different calibration solutions for standard curve of mixture of DZN were prepared in acetonitrile. The range of concentrations was between 200 to 6400 ng/mL. Linear calibration curves were acquired by drawing the peak areas of the different individual compounds. 

### Limits of detection and limits of quantitation

To measure the limits of detection (LODs), and limits of quantitation (LOQs), the lowest concentration was detected or quantitated, by considering 1:3 and 1:10 ratio of the baseline noise and calibration point, respectively. For the purpose of confirmation, the LOQ was performed five times for accuracy of the results.

### Real-time polymerase chain reaction (RT-PCR)

For Real-time PCR, 1 μg of total RNA was reverse transcribed to cDNA using a Primescript RT reagent kit (TAKARA, Japan). Real-time PCR was performed using the Step one plus ABI system (Applied Biosystems). RT-qPCR was performed under the following conditions: 30 s at 95 °C for 1 cycle, 40 cycles of 95 °C for 5 s, 60 °C for 34 s and 72 °C for 45 s. One cycle of 30 s 72 °C was done finally to allow final extension. All curves were linear in the range tested (R^2^ > 0.999). Based on the linear equation of standard curves, LOQ and LOD were calculated and invalid data were not included in the final analysis. The sequences of primers used for qPCR are listed in Table 1(Tab. 1).

### Statistical analysis

The results were presented as mean ± standard error of mean (SEM). One-way analysis of variance (ANOVA) and Tukey's multi-comparison tests were taken. The degree of significance was set at (P< 0.05). Also, in order to evaluate correlations, Pearson's test was used. 

## Results

### Effect of diazinon on plasma AChE activity

**Figure 1:**
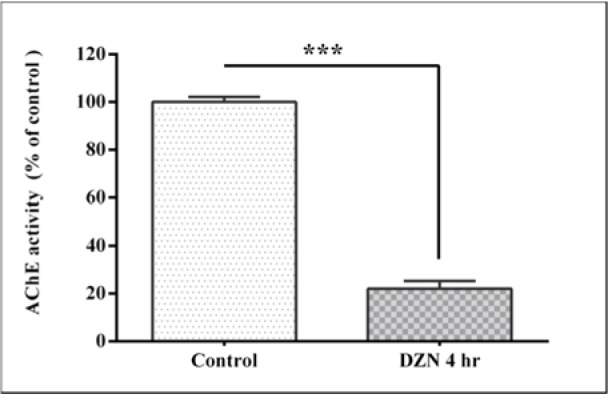
Effect of acute exposure to diazinon (DZN) on plasma acetyl cholinesterase (AChE) activity in terms of inhibition percentage of control in rat. The values are expressed as the percentage of control and are the mean ± SEM in six rats in each group. *** (P< 0.001) significantly different from control.

Plasma AChE activity is shown in Figure 1(Fig. 1). Administration of DZN at a dose of 85 mg/kg reduced AChE activity to 22.06 ± 5 % as compared to controls (P< 0.001). This shows an inhibition of the enzyme activity up to 77.94 %.

### Oxidative stress biomarkers

#### Lipid peroxidation (LPO) 

**Figure 2:**
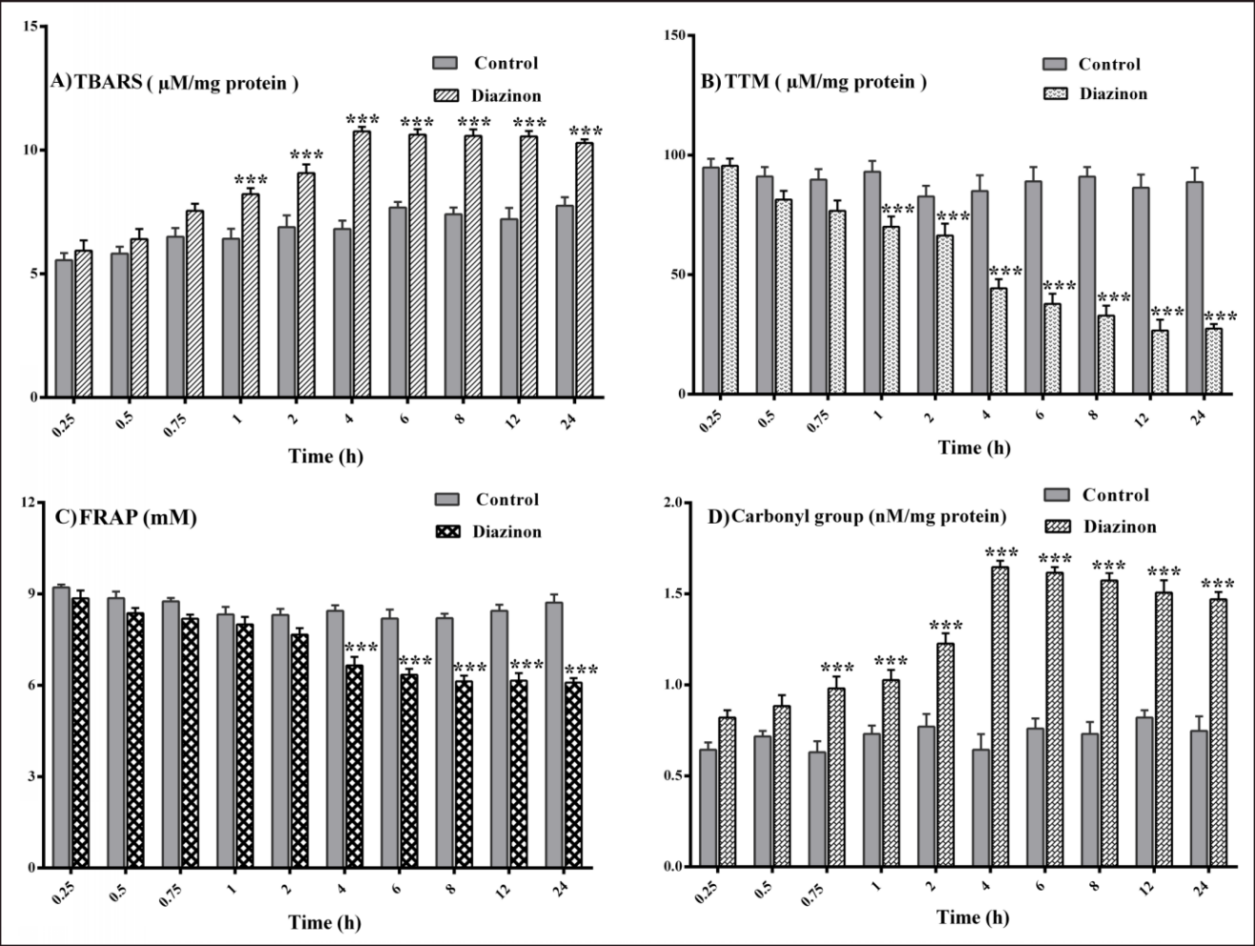
Changes in plasma levels of oxidative stress biomarkers after 0.25, 0.5, 0.75, 1, 2, 4, 6, 8, 12 and 24 hours of 85 mg/kg diazinon administration. Values are shown as mean ± SEM of six animals in each group, *** (P< 0.001) significantly different compared with control; (A) lipid peroxidation (TBARS), (B) total thiol molecules (TTM), (C) ferric-reducing antioxidant power (FRAP), (D) protein carbonyl.

A significant increase in the level of LPO in plasma was found after 1 hr when TBARS values in DZN groups were compared to the same time point of control groups (P< 0.001). At the same time, TBARS level did not significantly change in treated groups after 4 hrs (Figure 2A(Fig. 2)).

#### Total thiol molecules (TTM) 

The level of plasma TTM in DZN groups after 1 hr decreased significantly compared with the same time point of control groups (P< 0.001). There were no significant changes in the level of TTM in treated groups after 4 hrs (Figure 2B(Fig. 2)).

#### FRAP

Plasma FRAP in DZN groups after 4 hrs was decreased significantly compared with the same time point of control groups (P< 0.001). Significant changes in FRAP values were not found after 4 hrs in the treated groups (Figure 2C(Fig. 2)).

#### Protein carbonyl groups

As shown in Figure 2D(Fig. 2), the carbonyl groups, as a measure of protein oxidation increased significantly in the DZN groups after 0.75 hrs, when compared to the same time point of control groups (P<0.001). Similar to other oxidative stress biomarkers, protein carbonyl groups modifications were not statistically significant between DZN groups after 4 hrs. The effect of DZN on the levels of all oxidative stress biomarkers was not time-dependent (Figure 2D(Fig. 2)). 

### Correlation of changes of diazinon levels in plasma with oxidative stress biomarkers 

**Figure 3:**
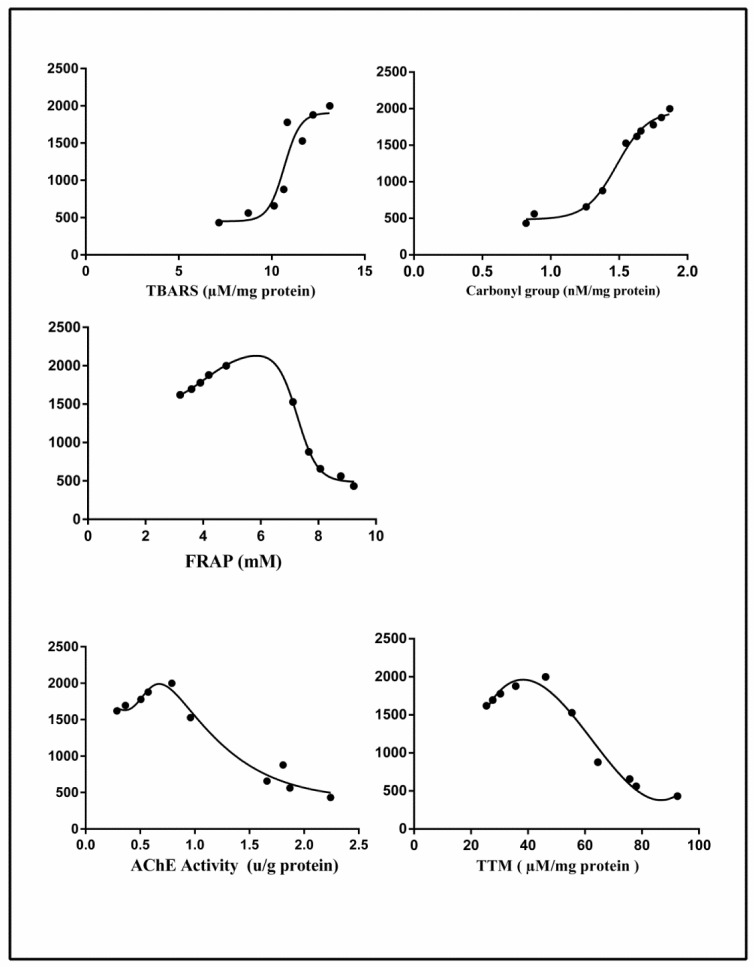
Correlation between plasma diazinon levels and oxidative stress markers

A significant negative correlation was found between the concentration of DZN in the plasma with AChE, FRAP and TTM respectively (r = -0.9266, P<0.0001, r = -0.8989, P<0.0004, r = -0.9013, P<0.0004). In contrast, a significant positive correlation was observed between the concentration of DZN in the plasma with TBARS and protein carbonyls, respectively (r = 0.8044, P< 0.0050, r = 0.9583, P<0.0001) (Figure 3(Fig. 3)). 

### Effects of diazinon on mRNA expression of PON1, CAT and GPx in liver tissue

**Figure 4:**
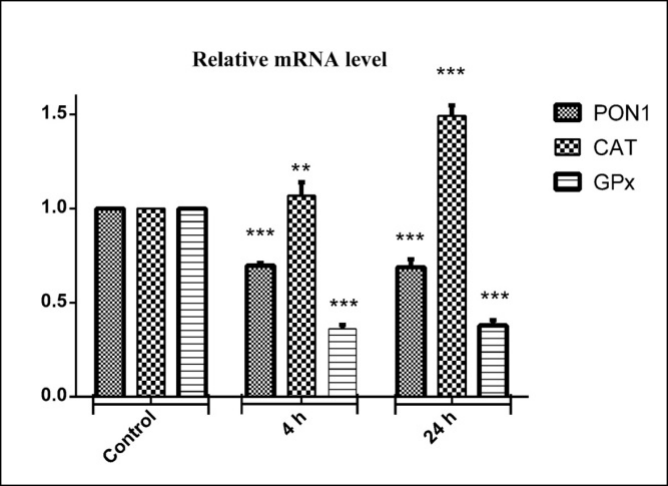
Effects of diazinon on mRNA expression pattern of PON1, CAT, GPx genes in liver tissues. Animals were exposed to 85 mg/kg diazinon orally, after treatment (4 and 24 hours); mRNA expression was measured with real time PCR. Data are expressed as means±SEM. ** (P< 0.01) and *** (P< 0.001) significantly different compared with control.

Figure 4(Fig. 4) shows the effect of DZN on expression of three candidate genes that encode antioxidant CAT and glutathione peroxidase (GPx) and (PON1) enzymes in liver tissue. At 4 and 24 hrs after DZN treatment, the change folds of mentioned genes were evaluated by Real-time PCR. GPx gene expression was significantly decreased in mRNA level by 2.8-fold (P<0.001), compared to the control group. Additionally, the PON1 gene expression level was significantly decreased in mRNA by 1.5-fold (P<0.001). Whereas, CAT gene expression was significantly increased in mRNA level by 1.1-fold in 4 hrs (P<0.01) and 1.5-fold (P<0.001) in 24 hrs after DZN exposure, compared to the control group.

### Histopathological examination in liver tissue 

**Figure 5:**
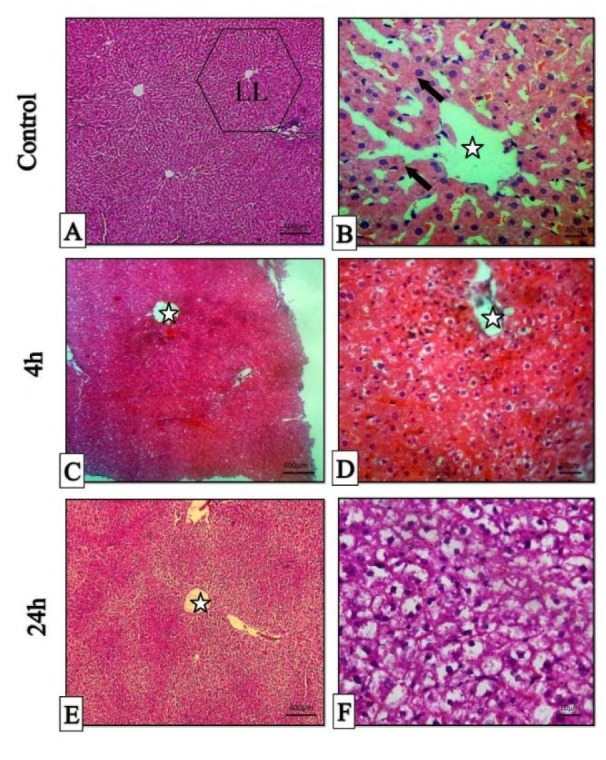
Histopathologic sections of liver, H&E stain, Stars: central veins, Arrows: hepatocyte cords, LL (classical liver lobule). A & B: histopathological sections of normal liver (Control), C & D: micrographs of liver 4 hours post-treatment. E & F: micrographs of liver 24 hours post-treatment.

**Figure 6:**
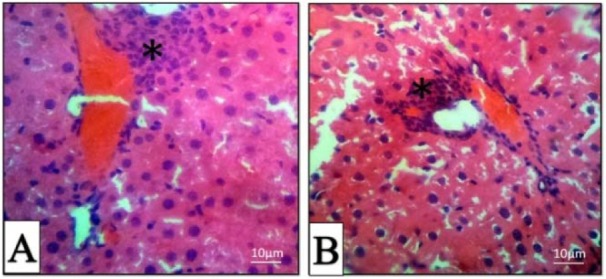
Histopathologic sections of the liver in 4 (A) and 24 (B) hours post-treatment, H & E stain. Mononuclear inflammatory cells (Star).

All H&E-stained liver sections from different experimental groups were evaluated histologically (Figure 5A-F(Fig. 5)). The histopathological evaluation of liver 4 hrs post-treatment showed mild to moderate hepatocyte swelling (hydropic degeneration) and the classical architecture of the liver has been deteriorating. Sinusoidal congestion has also occurred due to hepatic cells swelling (Figure 5C, D(Fig. 5)). Mononuclear inflammatory cells (MICs) in portal area (portal hepatitis) are seen in Figure 5A(Fig. 5). Histopathological evaluation of 24 hrs samples revealed severe hepatocyte cell swelling (ballooning degeneration) and hyperemia in sinusoidal spaces (Figure 5E, F(Fig. 5)). The assessment of the portal-triad area showed MICs portal hepatitis, just like the previous sample (Figure 6B(Fig. 6)). It seems that the toxic effect has been increased over time. Cellular degeneration, hyperemia and sinusoidal congestion have been increased in 24 hrs sample when compared to the sample which has been taken 4 hrs post-treatment. Overall, the toxic effect of this treatment has been proved by histopathological analysis.

### Determination of diazinon levels in plasma

**Figure 7:**
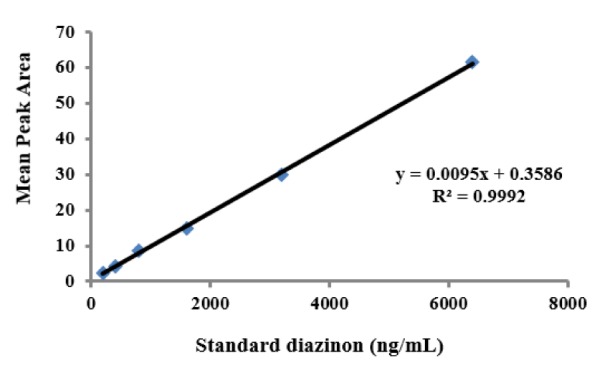
Standard calibration curves of diazinon

**Figure 8:**
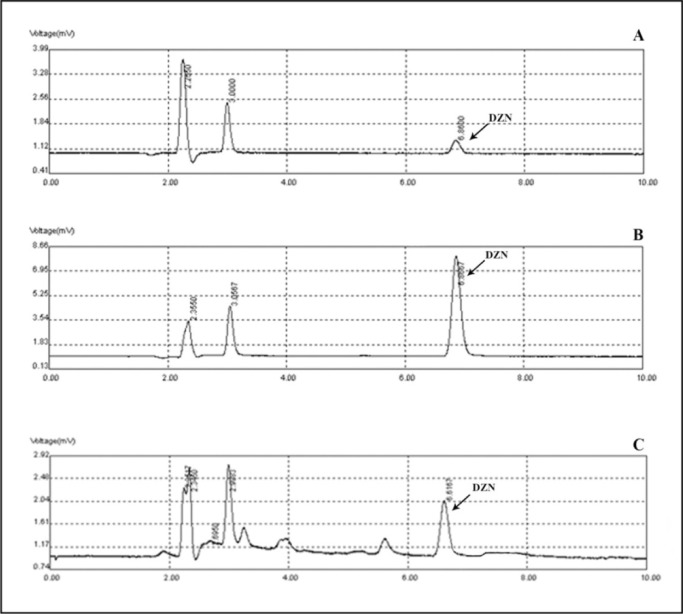
Chromatograms of diazinon in standard solutions of 400 ng/mL (A), 6400 ng/mL (B) and in rat plasma at 4 hours following gavage administration (C).

**Figure 9:**
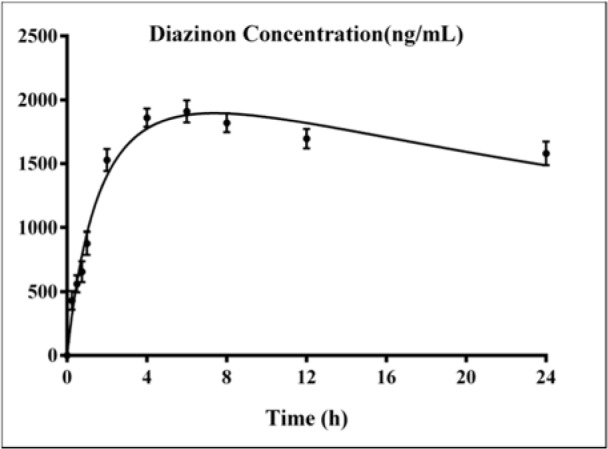
Variations of diazinon levels in rat plasma samples after 0.25, 0.5, 0.75, 1, 2, 4, 6, 8, 12 and 24 hours of DZN (85 mg/kg, p.o.) administration. Values are shown as mean ± SEM of six animals in each group.

The standard curves of the calibration of the peak area versus the concentrations of DZN are shown in Figure 7(Fig. 7). The calibration plot of the analyte was excellent and linear over the concentrations range of 200 to 6400 ng/mL, and the correlation coefficient values were 0.9992. The LOD (S/N= 3) and the LOQ (S/N= 9) were 137.42 and 416.52 ng/mL, respectively. The retention times and areas under the curve were approximately similar in all injections. The DZN samples were found at concentrations ranging from 200 to 6400 ng/mL. Spiked plasma samples after analyzing by Sep-Pak cartridges using HPLC conditions have been described previously for each concentration in three replicates. The average percentage of the plasma recoveries was 90.62 % to 95.72 %. Chromatograms of DZN in standard solutions of 400 ng/mL (A), 6400 ng/mL (B) and in rat plasma sample after 4 hrs of DZN administration are illustrated in Figure 8(Fig. 8). The plasma concentration of DZN was increased significantly at each time point until 4-hr, while considerable changes in plasma DZN levels were not found after this time point in treated rats (Figure 9(Fig. 9)). 

## Discussion

The toxicity of DZN is mainly due to the formation of its toxic metabolite, diazoxon, and its potential ability to cause AChE inhibition in the target tissues (Poet et al*.*, 2003[47]). Blood levels of DZN were actually quantified from the animals according to 85 mg/kg dose equal to 1/5th of LD_50_ and were used to evaluate the biochemical parameters (WHO, 2010[60]). It was observed that acute exposure to DZN is able to inhibit AChE and induce oxidative stress, indicating that the threshold level of DZN for induction of toxicity is very low. In addition, DZN caused changes in hepatic cells and its antioxidative gene expression. DZN was quantified at time-courses of 0.25, 0.5, 0.75, 1, 2, 4, 6, 8, 12 and 24 hrs post treatment. According to our results, 6 hrs after post treatment (85 mg/kg), the peak level of DZN in blood was obtained as 1910 ng/mL. The results of the current study indicate that DZN is rapidly absorbed after oral administration. It has been suggested that DZN's oral absorption is high and after a couple of hours, most of the substance is metabolized (Ogutcu et al*.*, 2006[41]). These data are almost similar to previous study indicating that blood levels of both DZN and 2-isopropyl-4-methyl-6-hydroxypyrimidine (IMHP, as major metabolites), reached peak levels after 6 hrs of treatment and show a linear kinetics of DZN and IMHP (Poet et al*.*, 2004[46]). 

DZN is well known and has toxic effects through inhibition of AChE activity in biological system. Oxidative stress is defined as a state of impaired balance between ROS production and antioxidant capacity, which may cause various harmful effects on cellular macromolecules such as DNA, lipids, and proteins (Saeidnia and Abdollahi, 2013[50]). Lipid peroxidation is mainly due to the overproduction of ROS by both exogenous and endogenous sources and is the most prominent event in the biological system which is linked to an extensive series of different biological processes (Mostafalou and Abdollahi, 2017[37]).

Our results demonstrated that the applied dosages of DZN, as used in our experiment could affect the antioxidant defense system and LPO level. MDA is a major oxidation product of peroxidized polyunsaturated fatty acids and increased concentration of MDA is one of the key indicators of lipid peroxidation (Pakzad et al*.*, 2013[45]). This is clear from our observation that, upon DZN treatment, the concentration of MDA and the amount of antioxidant defense level in plasma differs from the control. Induction of LPO occurred in a time-dependent manner by DZN and its level elevated in the early minutes after DZN exposure. Protein carbonyl content is actually considered the most general indicator for oxidative stress assay, and by far the most commonly used marker of protein oxidation, and we also found from the literature that accumulation of the protein carbonyls linked with several human diseases. The results of the present study showed that the protein carbonyl content in the plasma was significantly elevated at 0.75 hr after DZN administration. As a previous report, the protein carbonyl level changed more quickly in comparison to LPO (Dalle-Donne et al*.*, 2003[13]).

In physiologic conditions, antioxidant enzymes, including TTM and FRAP play an important role in the defense against ROS and mainly monitored for the evaluation of antioxidant system function (Pakzad et al*.*, 2013[45]). A considerable decrease in the FRAP level of the serum may be due to prevailing oxidative stress under the influence of ROS produced from DZN-induced oxidative stress. In the current study, plasma FRAP until 4 hrs showed no significant difference among all the treatment groups. However, within 4 hrs, FRAP levels of the tested groups were significantly decreased. The power of antioxidant enzyme activity after the first exposure to oxidizing agents significantly decreased with subsequent disruption of the antioxidant defense system (Doyotte et al*.*, 1997[14]). A delay in the reduction of FRAP level as compared to protein carbonyl groups and LPO was observed in our study, which explains the role of oxidative stress as the minimum consequence of DZN toxicity in biological system. Our results suggest that DZN rapidly targets lipids and proteins and triggers lipid and protein oxidation processes through the production of free radicals. At the same time, free radicals generated by ROS can cause a direct effect on the antioxidant enzymes and induce imbalance of oxidative/antioxidant reactions. In this condition, breakdown of products of LPO which are produced earlier, promotes antioxidant enzyme dysfunction mechanism and thus decrease the FRAP levels. Results of previous studies suggest that inhibition of AChE activity by OPs may occur as a consequence of accumulation of ROS and other oxidative agents. Our results showed that AChE activity was inhibited about 77.94 % in comparison to control in 4 hrs post-treatment of DZN. It possibly resulted in an increase in the production of ROS. For the time being, there are very limited studies on the relationship between antioxidant enzyme status and DZN plasma level in acute toxicities. However, it is well established that the liver, as a main organ responsible for metabolism and detoxification, plays a critical role in DZN poisoning (Teimouri et al*.*, 2006[58]; Amirkabirian et al*.*, 2007[4]; Gokcimen et al*.*, 2007[18]), so we investigated its effects on hepatocellular antioxidant system components, including PON1 and GP_X_. For this purpose, we assessed the relation of DZN plasma concentration and plasma levels of PON1 and GP_X_. PON1 is a calcium dependent esterase produced in the liver and released into the plasma. This enzyme plays several roles; among the most important ones are prevention of LDL oxidation and detoxification of some OP metabolites. DZN generates highly reactive free radicals, which react with sulfhydryl groups in some molecules such as glutathione and protein thiols (Jaouad et al., 2006[22]). The potential antioxidant activity of PON1 is associated with its -SH groups and changes in the nature and amount of free thiol groups in PON1 molecule might lead to the decrease in its antioxidant activity (Brattin et al*.*, 1985[11]). Thus, during an oxidative condition, the enzyme activity and its gene expression start to subside. Our data revealed that the oxidative stress can result in suppression of PON1 gene expression compared with the control group. Interestingly, the concurrent decrease in total antioxidant power and PON1 expression was also observed. Therefore, reduced total antioxidant enzyme activity contributes to the decrease of PON1 gene expression. Similar to our results, in an *in vitro* experiment, exposure of the HepG2 cells with OP pesticides decreased PON1 gene expression (Medina-Díaz et al*.*, 2017[32]). In another study, oxidative stress induced by CCl_4_ significantly reduced expression of PON1 and PON3 genes in liver tissue (Hafez et al*.*, 2014[19]). 

Antioxidant enzymes such as GPx, CAT and SOD have a prominent role in the ROS process during oxidative stress reactions. GPx is a necessary enzyme for reduction of hydroperoxides by glutathione (GSH). Catalase is a very powerful antioxidant enzyme, whereby 5 million molecules of hydrogen peroxide are converted to water and oxygen at every second (Wang and Hai, 2016[59]; Ponce-Ruiz et al*.*, 2017[48]). The current study showed 2.8-fold decrease in mRNA level of GPx gene in the liver tissues, compared to the control group, whereas gene expression of CAT significantly increased in a time-dependent manner. Unlike CAT, which is mainly involved in the destruction of hydrogen peroxide, GPx has a broader spectrum of antioxidant functions (Limaye et al*.*, 2003[27]). Reducing the expression of the PON1 and GPx genes was consistent with the oxidative stress indices. The plasma level of the oxidative biomarkers FRAP and TTM significantly decreased, whereas TBARS and carbonyl levels significantly increased in DZN group. The rational reason for the elevation of CAT gene expression after oxidative stress has already been described (Tacchini et al*.*, 1996[57]). Their results indicated that post-translational regulation is responsible for increased hepatic CAT gene expression after oxidative stress induced by nitrofurantoin. 

In the present study, histopathological observation showed that DZN induced mild to moderate hepatocyte swelling (hydrophic degeneration) within 4 hrs of the post-treatment. Histopathological evaluation of 24 hrs sample revealed severe hepatocyte cell swelling (ballooning degeneration) and hyperemia in sinusoidal spaces. The evaluation of the portal-triad area showed MICs portal hepatitis. It seems that the toxic effect of DZN increased over the period of time. Cellular degeneration, hyperemia and sinusoidal congestion increased in 24 hrs sample as compared to the samples taken over 4 hrs of the treatment. This result is supported by previous studies indicating the role of environmental toxicants in endocrine disruption. Moreover, evidence related to the FRAP, TTM, LPO and protein carbonyl levels along with the gene expression changes supports our current outcome (Maqbool et al*.*, 2016[29][30], 2017[31]). In a similar study, amylase and lipase serum levels were significantly increased within three hours after administration of 75 mg/kg of DZN (Dressel et al*.*, 1982[15]). 

We conclude that acute administration of DZN promotes MDA generation and malfunction of the plasma antioxidant systems in a time-dependent manner. Thus, DZN even at a non-lethal dose produces a toxic effect on the gene expression of antioxidant enzymes of the liver. Change of antioxidant defense system occurs prior to DZN plasma peak time. 

Evaluation of oxidative stress biomarkers as well as the gene expression changes of these enzymes can be used as an effective tool in management of DZN-induced acute toxicity.

## Acknowledgements

This study was in part supported by a grant from TUMS with code number 96-01-45-34625. We are also grateful to Maryam Baeeri and Mahdi Gholami, who kindly assisted the research.

## Author contributions

All authors have directly participated in the planning or drafting of the manuscript and have read and approved the final version.

## Conflict of interest

The authors declare no conflict of interest.

## Figures and Tables

**Table 1:**
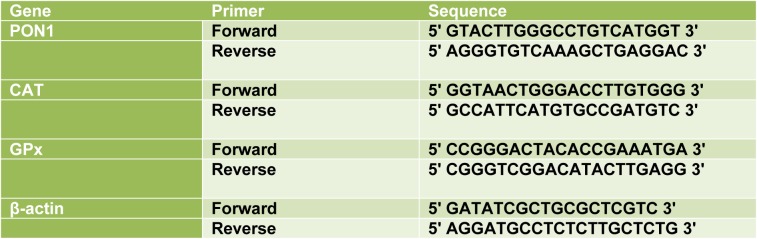
Sequences of different primers used for Real-Time PCR reactions
